# Osteochondral Allograft Transplantation of the Knee Using a Low-Cost Custom Hybrid Workstation Instrumentation: Technical Note with an Illustrative Case

**DOI:** 10.3390/jpm16040187

**Published:** 2026-03-30

**Authors:** Danijel Jurković, Stjepan Ivandić, Tomislav Čengić, Stipe Ćorluka

**Affiliations:** 1 Sestre Milosrdnice University Hospital Centre, 10000 Zagreb, Croatia; danijel.jurkovic@kbcsm.hr (D.J.); stjepan.ivandic@kbcsm.hr (S.I.); tomislav.cengic@kbcsm.hr (T.Č.); 2 University of Applied Health Sciences, 10000 Zagreb, Croatia; 3 School of Medicine, University of Zagreb, 10000 Zagreb, Croatia

**Keywords:** osteochondral allograft transplantation, workstation, osteochondral defect, cost reduction

## Abstract

Osteochondral allograft transplantation is a safe and effective surgical option for the treatment of large, focal, full-thickness chondral and osteochondral defects, particularly in young patients. We describe a low-cost new hybrid workstation for osteochondral allograft transplantation based on modified Ilizarov components and its clinical application in a patient with a large osteochondral femoral defect. The technique was applied in a 28-year-old male with chronic knee pain following two prior failed arthroscopic surgeries. Osteochondral allograft transplantation was performed using our modified workstation instrumentation. At the 8-month follow-up, MRI revealed excellent incorporation of the graft, and the patient reported ambulation without pain with return to physical activity. Our hybrid workstation presents a cost-effective alternative for graft preparation while maintaining a high standard of surgical care.

## 1. Introduction

The treatment of large osteochondral defects may pose a significant challenge for younger patients. These lesions, if symptomatic, can cause significant distress and greatly lower the patient’s quality of life, especially for those with high functional demands [1].

While there are a variety of surgical options for osteochondral defects (OCD)- such as microfracture, autologous chondrocyte implantation (ACI), and osteochondral autograft transfer system (OAT)—they may be inadequate for larger lesions [2]. On the other hand, other surgical options such as total knee arthroplasty are unsuitable for young individuals.

Osteochondral allograft transplantation is a possible alternative. It can address large osteochondral defects, and multiple studies have demonstrated its efficacy and safety [3,4].

Indications include large osteochondral lesions in both primary and revision settings. Significant advantages include the ability to cover defects of all sites and restore complex joint surfaces while avoiding donor site morbidity [5].

Absolute contraindications include advanced multicompartmental osteoarthritis and inflammatory arthropathies, while relative contraindications include smoking, alcohol abuse, chronic steroid use, ligamentous instability, uncorrected joint malalignment, obesity (BMI > 30 kg/m^2^), and the absence of >50% of the ipsilateral meniscus [6]. The two most commonly used surgical techniques are Dowell and Shell, which are chosen depending on the defect characteristics, with the latter being indicated for large and irregular defects [5]. In the Dowel technique, an osteochondral donor plug is extracted using one of several osteochondral instruments and workstations and is sized to match the corresponding recipient site [5]. Several commercially available workstations are available on the market, including the Arthrex Allograft OATS Instrument Set and the JRF Ortho Osteochondral Allograft Plug Instrumentation. Different sets usually consist of instrumentation used for the preparation of the recipient site and a workstation that holds the raw donor allograft while the preparation is taking place. Commercial workstations vary in design depending on the company, but they usually consist of a holder that seats the graft and a securing mechanism that ensures that the graft stays firmly in place during preparation. Instrumentation sets and workstations can be acquired separately. Subsequently, a significant reduction in cost is achievable if one of the two is omitted. As workstation requirements are rather simple compared to the precise instrumentation required for graft preparation (holding the graft firmly and securely in place), a simple workstation can be designed using readily available resources. In this technical report, we describe the design and clinical application of a novel custom-made workstation for osteochondral allograft preparation based on a modified Ilizarov frame. A commercially available system (Arthrex, Naples, FL, USA) in combination with our own custom-designed graft preparation workstation was used to prepare the host defect and donor graft for implantation, forming what we term the hybrid instrumentation. The main benefit of our hybrid instrumentation is its significantly reduced cost, as our workstation can be assembled from resources readily available at every traumatology department. As the two most significant downsides of osteochondral allograft transplantation surgery include costs and graft availability, our workstation design aims to significantly reduce surgery-related costs without sacrificing standards of care.

## 2. Workstation Design

**Figure 1:**
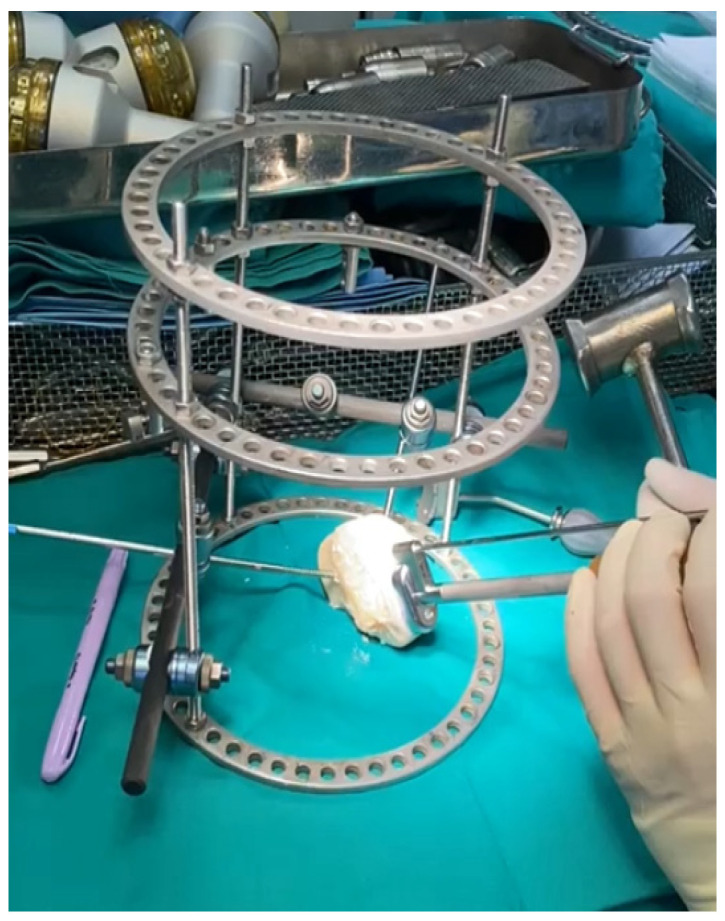
Hybrid Ilizarov external fixator workstation with fixed osteochondral allograft.

Several features of the appropriate workstations include graft seating, a mechanism that stabilizes the graft in place, and an overarching superstructure. In pursuit of maximal simplicity, we chose not to include a designated graft seat, focusing instead on an adequate graft fixation mechanism. The fixation mechanism in the case of pins or wires needs at least two points of anchorage, preferably more. The superstructure needs to be sufficiently stable to neutralize the forces acting on the fixation mechanism. This superstructure can be conceptualized as a frame or a kind of foundation upon which the rest of the mechanism is laid. Having set requirements and aims of our workstation and taking into consideration materials and tools available to us, we have decided on using Schanz pins, Ilizarov wires, as well as Ilizarov frames and external fixator carbon rods. We designed our own graft workstation as follows: three Ilizarov rings were connected with three steel rods, leaving one quarter of a ring diameter between the bottom two rings free as a “work window” (Figure 1). The original workstation design featured four steel rods for added stability. However, one rod was later omitted to increase the working window and improve visualization during graft preparation. Afterwards, two carbon rods were connected to steel rods using clamps, and on each of the carbon rods, one Schanz pin was placed using clamps. The Schanz pins were used to fix the allograft in place during graft preparation. We initially intended to use at least three points of fixation. Several different Schanz pin and Ilizarov wire configurations were assessed. These include three Schanz pins, two pins with one, two, or three wires, as well as three pins with a variable number of wires. We have come to the conclusion that two Schanz pins provide sufficient stability while avoiding clutter. Pins were placed at a 90-degree angle in the transverse axis and approximately 45 degrees of sagittal inclination. This configuration sufficiently resisted multidirectional forces acting on the graft during preparation and hammering. We recommend having more pins and wires sterilized and ready so that they can be added to carbon rods to provide additional stability if required. Our set can be easily assembled, disassembled, and sterilized for multiple uses. Its modular design is of great advantage as it is significantly less bulky than commercially available sets, which eases logistics and lessens transportation issues. The absence of a designated graft seat is intentional. When the graft is secured in place with pins, it is level with the table, and this table provides a third point of stabilization, a counter force to Schanz pins. We have hypothesized that the third point of fixation should be separate from the workstation itself, as this reduces the force transmitted via the hypothetical graft seat through the frame and into the pins. Using the table as an anchor point allows for energy dissipation outside of our workstation.

## 3. Case Description

**Figure 2:**
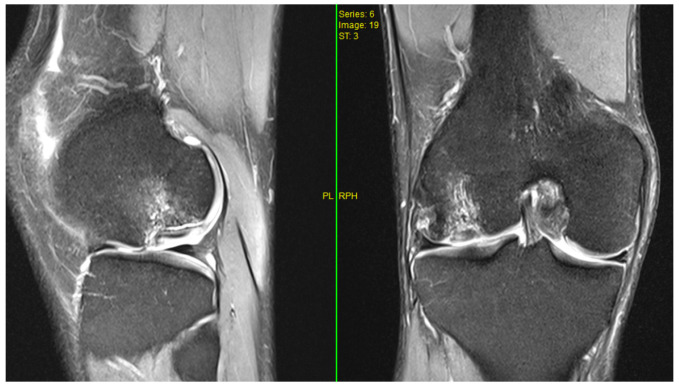
Preoperative MRI of the right knee with an osteochondral defect.

A healthy 28-year-old man presented to our clinic with right knee pain. On clinical examination, he had an antalgic gait and reported moderate, intermittent pain, especially during flexion. He lacked the terminal 30° of flexion and experienced catching and locking during work and everyday activities. His Tegner–Lysholm Knee Score was 59. Radiographs showed an osteochondral defect of the posterior aspect of the lateral femoral condyle with a detached fragment. MRI demonstrated an osteochondral fragment measuring 3 × 2 cm, without displacement (Figure 2).

His history included arthroscopic fixation using chondral darts. After surgery, he was pain-free for 8 months. Subsequently, the pain recurred, and he underwent arthroscopic debridement and microfracture. Six months after the second procedure, there was no improvement in symptoms.

Eight months after the debridement and microfracture, the patient still had symptoms despite structured physiotherapy and NSAIDs, so we decided to proceed with a fresh-frozen osteochondral allograft. We chose this option because the defect was large (>2 cm), the patient was young (<30 years), and we needed a salvage solution. A fresh-frozen osteochondral allograft measuring 37 mm (mediolateral) × 65 mm (anteroposterior) was obtained from a donor who met all criteria, in collaboration with the Barcelona Tissue Bank.

## 4. Surgical Procedure

The surgical procedure was performed with the patient in the supine position with a tourniquet on the proximal ipsilateral thigh. A lateral parapatellar approach was used, and the lateral condyle, along with the osteochondral defect, was exposed.

The osteochondral defect was measured, and a matching sizer from the BioUni OATS Arthrex set was selected and applied to the lateral condyle. The planned grafting area was then marked. The fresh-frozen osteochondral allograft was secured in our custom hybrid Ilizarov workstation (Figure 1) using two Schanz pins. The exact harvest location was marked according to the previously chosen sizer.

**Figure 3:**
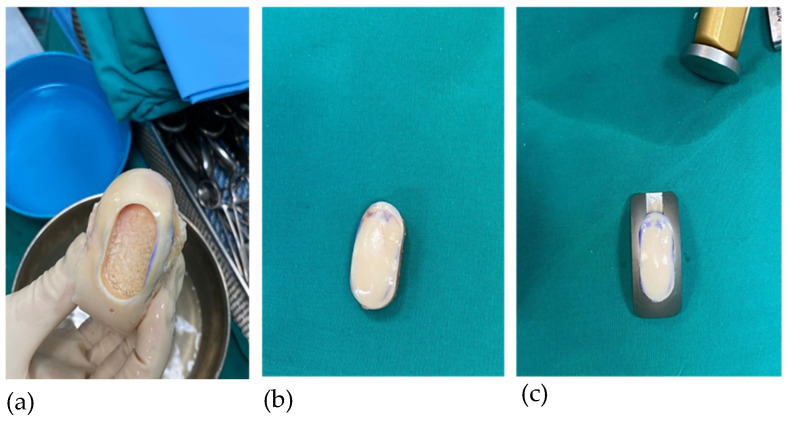
Osteochondral allograft; (**a**) final cut of the replacement cartilage; (**b**) newly shaped graft; (**c**) graft in donor trial.

The sizer was applied to the allograft surface and advanced to identify a flush fit. The oblong cutter was placed over the marked area and fixed with a Steinman pin. Afterwards, the assembly was advanced into the allograft cartilage and bone to the appropriate depth using the impact handler. The saw depth guide and sagittal guide were assembled and placed into the previous oblong cutter cut. The assembly was then advanced until properly seated at the predetermined depth. The final cut of the replacement cartilage was created with the sagittal saw (Figure 3). The new elliptically shaped cartilage allograft was recovered from a saw depth guide, soaked with autologous conditioned plasma (ACP, Angel Arthrex system), and prepared for implantation.

**Figure 4:**
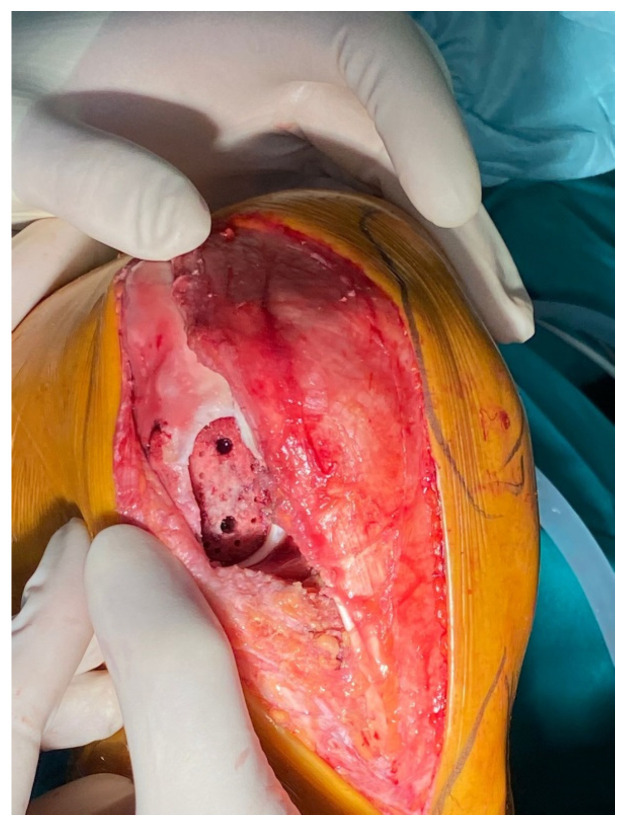
Socket preparation of the lateral femoral condyle.

Next, the previously chosen sizer was positioned over the osteochondral defect, and two drill pins were inserted through the guide holes. A scoring device was advanced over two drill pins and tapped into the cartilage to create perforations, followed by the creation of an oblong socket matching the dimensions of the allograft. Two sockets were created with the drill depth guide and reamer. The box cutter was advanced over the drill pins to the bottom of the socket, and the remaining fragments were removed. Nanofractures were created to generate a bleeding subchondral bed to enhance graft incorporation. (Figure 4).

**Figure 5:**
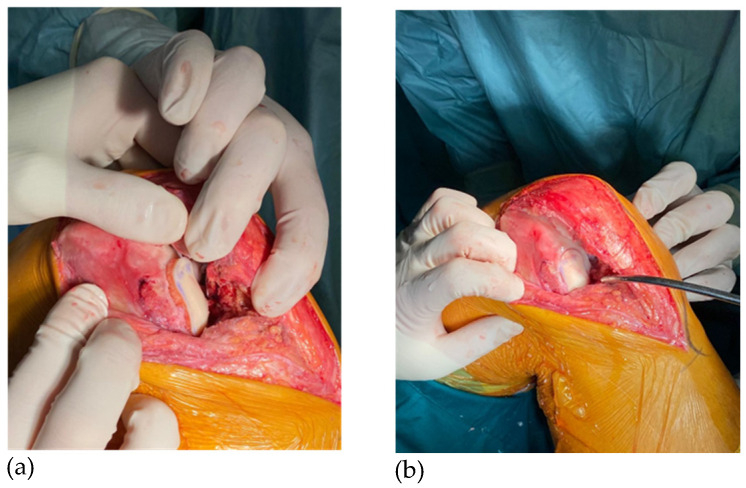
(**a**) Allograft implantation; (**b**) Allograft flush with surrounding cartilage using a tamp.

Finally, the replacement cartilage allograft was advanced into the socket until flush with the surrounding cartilage (Figure 5). No perioperative or graft-related complications occurred.

## 5. Postoperative Rehabilitation

The patient was allowed to begin toe-touch weight bearing with crutches immediately after surgery. His knee was immobilized in a hinged knee brace. A passive motion machine was used for the first 4 weeks. Closed-chain exercises, such as cycling, were introduced between weeks 6 and 8. Full weight bearing was allowed at 8 weeks postoperatively. Tegner and Lysholm scores at 3 and 6 months were 67 and 88, respectively. The patient was allowed to return to recreational and sports activities by 7 months.

## 6. MRI Assessment

**Figure 6:**
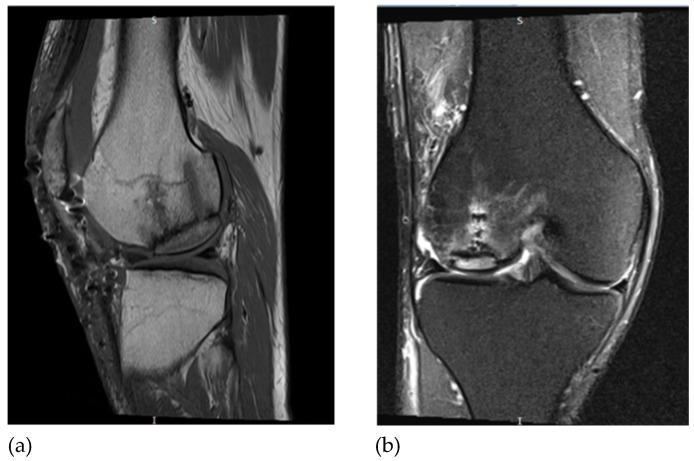
Postoperative knee MRI; (**a**) sagittal view; (**b**) coronal view.

At follow-up after 8 months of surgery, MRI showed osseous integration into the recipient bone, flush with the adjacent cartilage (Figure 6).

## 7. Discussion

Osteochondral allograft transplantation is a safe and effective surgical option suitable for a wide variety of osteochondral lesions [7]. Patients are usually young with large focal full-thickness chondral and osteochondral defects (>2 cm^2^) in the femorotibial and patellofemoral joint, osteonecrosis, and as a revision procedure after primary failed cartilage repair [7]. Alignment plays an important role in osteochondral reconstruction. Persistent malalignment may lead to compartment overload and compromise graft longevity. In the present case, the preoperative valgus was corrected to near-neutral on postoperative imaging; therefore, we focused on restoring the defect and joint surface rather than deliberate varus correction, which could lead to medial-side overload along with additional osteotomy-related morbidity. Although allograft transplantation is deemed safe and effective, with good to excellent outcomes, two important drawbacks are frequently cited: graft availability and high cost [8]. While the high cost of the procedure can be partially attributed to graft acquisition and associated logistical complexities, it is also related to specific required instrumentation sets. These costs can be partially mitigated by using custom-made instrumentation. While instruments used for precise graft preparation are highly specific and technically advanced, a graft workstation, whose sole purpose is holding osteochondral bone in place, can be made using available materials. Our workstation is designed as outlined in the corresponding chapter. The most important benefits include easy assembly and a modular design. The basic construct can be modified according to the specific surgeon’s needs. For example, although two Schanz pins provided adequate stability, an additional posterior pin may be added if required. Additionally, Ilizarov half rings can be added to the bottom of the construct at the point of contact with the table to provide additional stability. Compared to commercial workstations, it offers greater versatility and increased utility as it can be customized to meet specific surgeon needs. Its fundamental design remains highly modifiable, modular, and widely available. Furthermore, our workstation eliminates the need for an additional specialized commercial graft preparation station. Unlike commercial workstations, which are bulky and used infrequently and solely for one purpose, our workstation is versatile and can be disassembled and repurposed, which minimizes storage and space requirements. While precise quantitative assessment cannot be provided as it varies by region, our design allows for the omission of a commercial workstation, thereby representing a substantial reduction in required equipment. Potential limitations of our workstation include the need for an assistant to provide stability for the construct, as it is not secured to the table. Also, placing Schanz pins in a non-perpendicular direction may lead to a cutout. In these cases, it may be advisable to use Ilizarov wires for additional stability. The authors nevertheless believe our workstation could adequately address any osteochondral graft transplantation scenario, which includes graft preparation using a fixed workstation. Authors also believe that this design can be further tailored without limitations to suit any specific needs while retaining its basic design with Ilizarov frame and Schanz pins. The key advantage of using the Ilizarov frame is its modularity and high degree of customizability. Ultimately, a non-inferiority trial may further elucidate the efficacy and cost-effectiveness of our workstation compared to commercially available alternatives.

## 8. Conclusions

Osteochondral allograft transplantation is a reliable treatment in the younger population for large osteochondral defects with favorable graft survivorship. Our workstation design has shown promising early results. To our knowledge, this is the first technical report describing osteochondral allograft transplantation using a custom-built allograft preparation workstation. This intervention allowed for a significant reduction in costs while maintaining a high standard of surgical care. Although results are promising, more studies are required in order to fully validate the efficacy of this workstation.

## Data Availability

Data are contained within the article. Additional information is available from the corresponding author upon reasonable request.
